# Regulatory Effects of Acupuncture on Emotional Disorders in Patients With Menstrual Migraine Without Aura: A Resting-State fMRI Study

**DOI:** 10.3389/fnins.2021.726505

**Published:** 2021-10-04

**Authors:** Yutong Zhang, Ziwen Wang, Jiarong Du, Jixin Liu, Tao Xu, Xiao Wang, Mingsheng Sun, Yi Wen, Dehua Li, Huaqiang Liao, Yu Zhao, Ling Zhao

**Affiliations:** ^1^College of Acupuncture, Moxibustion and Tuina, Chengdu University of Traditional Chinese Medicine, Chengdu, China; ^2^Clinical Research Center for Acupuncture and Moxibustion in Sichuan province, Chengdu, China; ^3^Sichuan Province Building Hospital, Chengdu, China; ^4^Center for Brain Imaging, School of Life Sciences and Technology, Xidian University, Xi’an, China; ^5^Hospital of Chengdu University of Traditional Chinese Medicine, Chengdu, China; ^6^Chengdu Integrated Traditional Chinese Medicine and Western Medicine Hospital, Chengdu, China

**Keywords:** acupuncture, menstrual migraine without aura, emotional disorders, pain, fMRI

## Abstract

**Background:** Menstrual migraine without aura (MMoA) refers to a specific type of migraine that is associated with the female ovarian cycle. It is particularly serious and has brought huge life pressure and mental burden to female patients. Acupuncture has been commonly used to prevent migraines and relieve concomitant emotional disorders; however, the physiological mechanism underlying this intervention remains unclear. This study aimed to use resting-state functional magnetic resonance imaging (rsfMRI) to investigate whether acupuncture can modulate brain function and if the potential influence on brain activity correlates with improving emotional symptoms in MMoA patients.

**Methods:** Overall, 44 patients were randomly divided into a true acupuncture (TA) group and the sham acupuncture (SA) group. Patients underwent rsfMRI before and after 3-month treatment, the amplitude of low-frequency fuctuations (ALFF) and regional homogeneity (ReHo) in rsfMRI were calculated. Zung self-rating anxiety scale (SAS), Zung self-rating depression scale (SDS), frequency of migraine attacks, visual analog scale, and intensity of the migraine were used for evaluate the clinical effect. The clinical changes of variables were also used to further assess the correlation with brain activity in MMoA patients.

**Results:** After acupuncture treatment, the emotional symptoms of both groups of patients improved, and the clinical symptoms of migraine were alleviated. The major finding of our study was that patients with MMoA showed lower ALFF value in the left anterior cingulate and the value was positively correlated with the decreases in the SAS and SDS scores. In the SA group, common brain regions responded both in ALFF and regional homogeneity values mainly in the insula, and no significant correlations were observed between brain regions and clinical variables.

**Conclusions:** These results indicated that both two acupuncture treatments were helpful in treating migraine and could improve emotion symptoms. TA had a relatively better effect in reducing the frequency of migraine attack than SA. The two therapies have different modulation effects as TA regulates emotional disorders by modulating the frontal-limbic regions, and SA may modulate pain perception through the placebo effect on insula and by indirectly regulating emotional disorders. These findings provided evidence that acupuncture is a complementary and alternative therapy to relieve clinical symptoms in female patients with migraines and could help enhance clinical diagnosis and treatment.

**Clinical Trial Registration:** [http://www.chictr.org.cn/index.aspx], identifier [ChiCTR-IOR-15006648. Registered 23 June 2015].

## Introduction

Migraine is a common neurological disorder and is considered to be the second-largest contributor to worldwide disability (Karikari et al., 2018). More than 50% of female patients with migraines report that their symptoms are associated with menstruation, and the proportion of female migraineurs is two to three times that of male migraineurs (Vetvik and MacGregor, 2017). According to the third edition of the International Classification of Headache Disorders (ICHD-3), women who report that their migraines are associated with their menses are said to have menstrual migraine without aura (MMoA) (PavloviÄ‡ et al., 2015). Attacks during menstruation tend to be longer and are accompanied by more severe nausea than attacks that occur outside the menstrual cycle (No Author, 2018).

The high prevalence of migraine-related emotional disorders, such as depression and anxiety, is often overlooked. An epidemiological study suggested that patients with chronic migraine had a higher risk of major depression, anxiety, or suicidal behavior than subjects without migraine (Trejo-Gabriel-Galan et al., 2018). MMoA as a special subtype of migraine is more likely to induce or exacerbate many symptoms associated with various emotional disorders that are affected by the cyclic rise and fall of estrogen (Warnock et al., 2017). The coexistence of emotional disorders and migraines alters the quality of life and increases the burden and disability associated with migraine (Liao et al., 2020). However, specific therapeutic guidelines for depression and anxiety in migraine are unavailable. The use of psychoactive drugs increases headache frequency and susceptibility to medication overuse and disability (Pompili et al., 2009; Peck et al., 2015). Conversely, caution is needed regarding the use of migraine preventive drugs, such as flunarizine and beta-blockers, as they may be contraindicated in the presence of emotional disorders (Asmundson and Katz, 2009). Therefore, using non-pharmacological treatment strategies aimed to manage both migraine and comorbid psychiatric disorders are essential.

Acupuncture, a treatment modality of traditional Chinese medicine (TCM), has a long history in China and is increasingly being adopted in the West as a complementary and alternative preventive treatment for migraine attacks and to relieve pain. The latest randomized clinical trials (RCTs) suggest that acupuncture is an effective therapy for reducing the severity of pain and prevention of frequent attacks in migraineurs (Wang et al., 2015; Zhao et al., 2017; Xu et al., 2020). Furthermore, a systematic review showed that acupuncture is superior to analgesic medications in alleviating average headache intensity in patients with MMoA (Yang et al., 2020). Studies have also shown that acupuncture is efficacious for various types of depressive disorders (Smith et al., 2018). A retrospective cohort study proved that acupuncture could reduce the risk of depression and anxiety during the long-term follow-up period in patients with migraine (Liao et al., 2020). However, the potential regulatory effects behind the beneficial effects of acupuncture on MMoA have not yet been fully elucidated. In light of the risk of comorbid disorders, an investigation of the neural mechanisms behind acupuncture effects may aid in the development of effective treatment options that can be applied clinically.

Neuroimaging approaches, which provide feasible, efficient, and non-invasive tools for investigating the central mechanisms of acupuncture and are a global trend in acupuncture research (Zhao et al., 2014), have been used to measure structural and functional brain changes in patients with migraine (Schwedt and Dodick, 2009). In this study, the amplitude of low-frequency fluctuations (ALFFs) and regional homogeneity (ReHo) methods were used to compare the blood oxygen level-dependent (BOLD) signals in the brains of patients with MMoA during the resting-state. ALFF and ReHo methods are two important methods for depicting the various characteristics of resting-state fMRI signals. ALFF measures the intensity of neural activity at the single-voxel level, while ReHo measures the neural synchronization of a given voxel with its neighboring voxels (Cui et al., 2014). ALFF can directly demonstrate the BOLD signal and reflect spontaneous fluctuations in the voxels under the resting state (Zang et al., 2007). ReHo is based on a data-driven approach and thus requires no prior knowledge and has good test-retest reliability (Zuo et al., 2013). ALFF and ReHo analysis has been used to study migraine in our previous study (Liu et al., 2015), and other diseases like premenstrual syndrome (Pang et al., 2020), Alzheimer’s disease (Li et al., 2020a), and Parkinson’s disease (Shen et al., 2020), among others.

Hence, we hypothesized that, compared to that of sham acupuncture, the true acupuncture modulates emotion-affected dysfunctional brain regions in MMoA patients. In this study, we aimed to use rsfMRI to investigate whether acupuncture can modulate brain function and if the potential influence on brain activity correlates with improving emotional symptoms in MMoA patients.

## Materials and Methods

This trial was performed at the Hospital of Chengdu University of TCM. The study was registered with the Chinese Clinical Trial Registry (Identifier: ChiCTR-IOR-15006648), performed in accordance with the principles of the Declaration of Helsinki, and approved by the ethics committee at the Hospital of Chengdu University of TCM. The sample size calculation of a neuroimaging study is different from that of a classic randomized controlled clinical trial. Power analyses for neuroimaging studies rely on assumptions about BOLD signal amplitude, smoothness, brain location, and other factors that render principled *priori* designations difficult (Yu et al., 2020). According to a previous neuroimaging study (Desmond and Glover, 2002), a minimum sample size (*n* = 20) should be used in order to obtain 80% power with an error threshold of 0.002 at a single-voxel level. Considering a conservative dropout rate of 20% and incomplete data due to severe head movement during the MRI scan, the sample size in this study was increased to 25 participants per group; therefore, a sample size of 50 patients with MMOA was determined.

### Participants

Fifty eligible patients were equally allocated into the TA group and SA group (25 in each group). One patient in the TA group and five patients in the SA group dropped out during the study because of non-compliance with treatment schedule or inability to be contacted. In total, 44 patients who received acupuncture therapies were included in the final analysis of clinical outcome measures. All participants provided written, informed consent to participate after the experimental procedures were fully explained, and they were informed that they could stop participating at any time. All patients were enrolled from the outpatient clinic of the Departments of Neurology and Gynecology in two clinical centers: (1) the Hospital of Chengdu University of TCM; and (2) Chengdu Integrated TCM & Western Medicine Hospital. Recruitment took place from July 2015 to August 2018. The diagnosis of MMoA was established according to the ICHD-3 beta criteria (No Author, 2013). Participants who met all the following inclusion criteria were included in the study: (1) female, 18 -50 years of age, right-handed; (2) migraine attacks outside of the menstrual cycle did not exceed six per month; (3) a history MMoA for 6 months or more; (4) a stable 28 (±7)-day menstrual cycle; and (5) Zung self-rating anxiety scale (SAS)/Zung self-rating depression scale (SDS) score >50. Patients with any of the following conditions were excluded: (1) neurological diseases, immunodeficiency, bleeding disorders, or allergies; (2) MRI contraindications, such as claustrophobia; (3) any prophylactic headache medication or any acupuncture treatment during the last 3 months; alcohol or drug abuse; (4) pregnancy, lactation, or plans to become pregnant within 6 months; (5) Zung self-rating anxiety/depression scale score >50; and (6) psychiatric disorders, such as schizoaffective disorder, schizophrenia, organic mental disorder, psychotic features coordinated or uncoordinated with mood or bipolar disorder.

### Study Design

The total observation period in this study was three menstrual cycles for each patient. Patients who met the inclusion criteria were randomly assigned to the true acupuncture (TA) and sham acupuncture (SA) groups in a 1:1 ratio. All patients were asked to document their symptoms in headache diaries, and the outcome measurement was completed both for the baseline and 4, 8, and 12 weeks after randomization. Additionally, each patient underwent functional magnetic resonance imaging (fMRI) examinations at the baseline and at end of the treatment period. After scanning, all participants reported that they had not experienced any headaches or migraines and remained awake during the procedure.

### Intervention

In this study, traditional Chinese acupuncture was used and treatments were manipulated by two specialized acupuncturists with at least 5 years of training and 3 years of experience. They implemented acupuncture therapy in both groups in turns. The acupoints were selected according to traditional classic and systematic reviews of ancient and modern literature of acupuncture for MMoA upon several consensus meetings with experts based on experiences from our previous study (Wang et al., 2018). Patients in the TA group were given treatments that used the following acupoints: GB20 (Fengchi), GB8 (Shuaigu), PC6 (Neiguan), SP6 (Sanyinjiao), and LR3 (Taichong). The non-acupoints chosen were used in our previous studies for migraine prophylaxis (Zhao et al., 2017; Table 1).

**TABLE 1:** Details of the acupoint and non-acupoint groups.

Group	Acupoint	Manipulation
True Acupuncture	(i) Fengchi (GB20)	(i) is punctured obliquely 0.8 to 1.2 cm toward to apex nasi
	(ii) Shuaigu (GB8)	(ii) is punctured horizontally 0.5 to 0.8 cm
	(iii) Neiguan (PC6)	(iii) is punctured perpendicularly 0.5 to 1 cm
	(iv) Sanyinjiao (SP6)	(iv) is punctured perpendicularly 0.5 to 1.5 cm
	(v) Taichong (LR3)	(v) is punctured perpendicularly 0.5 to 1 cm
Sham Acupuncture	(i) At the medial arm on the anterior border of the insertion of the deltoid muscle at the junction of the deltoid and biceps muscles	(i) is punctured perpendicularly 0.5 to 1 cm
	(ii) The inside of the mid-thigh region 2 cm lateral to half the distance from the anterior superior iliac spine to the lateral superior corner of the patella on the rectus femoris	(ii) is punctured perpendicularly 0.5 to 1 cm
	(iii) The edge of the tibia 1 to 2 cm lateral to the Zusanli (ST 36) point horizontally	(iii) is punctured perpendicularly 0.5 to 1 cm
	(iv) Halfway between the tip of the elbow and the axillae	(iv) is punctured perpendicularly 0.5 to 1 cm
	(v) Halfway between the epicondylus medialis of the humerus and ulnar side of the wrist bilaterally	(v) is punctured perpendicularly 0.5 to 1 cm

All acupoints were punctured bilaterally using single-use stainless steel filiform needles (Hwato Needles, Sino-foreign Joint Venture Suzhou Hua Tuo Medical Instruments Co., China), 25–40 mm in length and 0.25–0.30 mm in diameter.

The depths of the inserted needles differed but were approximately 0.5–1.5 cm. Needles were twisted and rotated (90°< amplitude < 180°) at a frequency of 1–2 Hz. Stimulation was repeated one to three times to achieve the Deqi sensation (a sensation of soreness, numbness, distention, or radiating that indicates effective needling) in the TA group; subjects in the SA group did not experience Deqi. Each group’s treatment consisted of 27 or 27 ± 6 sessions according to changes in their menstrual cycle for 28 days or 28 ± 7 days, and each session lasted for 30 min. Interventions were performed 1 week before menses, once every other day for a total of three treatments. A total of two treatments were administered at the onset of menses and twice a week for the rest of the time (Table 2).

**TABLE 2:** Intervention sessions of the two groups.

Intervention (One menstrual cycle)	Before menses (1 week)	Onset of menses (1 week)	After menses (2 weeks)	Total treatments
Session	Once every other day of three times/week	Twice a week	Twice a week	9 ± 2 times

### Outcome Measures in Clinical Efficacy

The clinical outcomes were measured every 4 weeks, and the measurement parameters included SAS, SDS, the frequency of migraine attacks (defined as the number of migraine separated by pain free intervals of at least 48 h), visual analog scale (VAS) score of 0–10, and the intensity of migraines on a scale of 0–3. All patients were required to keep a headache diary records every 4 weeks after inclusion. The headache diary documented the time of migraine onset, duration, frequency, and severity (evaluated using the VAS).

### Functional Magnetic Resonance Imaging Data Acquisition

Magnetic resonance imaging was performed during the periovulatory phase (days 12–16 of the menstrual cycle). All patients with MMoA were migraine-free for at least 72 h at the time of the MRI scan. MRI data were acquired using a GE Discovery MR750 3.0 T system with an eight-channel, phased-array head coil (General Electric, Milwaukee, WI, United States). The functional images were obtained with a single-shot gradient-echo echoplanar imaging sequence with the following parameters: repetition time = 2,000 ms; echo time = 25 ms; flip angle = 90°, field of view = 240 mm × 240 mm, data matrix = 64 × 64, slice thickness = 3 mm, and voxel-size = 3.44 mm × 3.44 mm × 4 mm. During the whole functional scan, all participants were instructed to keep their eyes closed and stay awake during the entire session.

### Data Analysis

#### Clinical Data Analysis

SPSS version 23.0 software (SPSS, Chicago, IL, United States) was used for demographic analysis. The independent-sample *t*-test was used to compare all demographic characteristics between the two groups. *P*-values less than 0.05 false discovery rate (FDR) were considered to be statistically significant. Continuous variables were presented as means (standard deviation) with 95% confidence interval (CI). For continuous variables, a paired-*t* test was applied for within-group comparisons, two-sample *t*-tests were applied for two-group comparisons. Treatment effects including SAS, SDS, VAS, frequency of migraine attack every 4 weeks, and intensity of migraines every 4 weeks were evaluated using a repeated-measures analysis of variance (ANOVA) model with a between-subjects factor THERAPY (levels: TA and SA) and a within-subjects repeated measures factor TIME (levels: baseline, 1–4, 5–8, and 9–12 weeks). *P* value < 0.05 was considered as statistically significant.

#### Magnetic Resonance Imaging Data Preprocessing

The ALFF value for each voxel was calculated by taking the average of the square root of the power spectrum from 0.01–0.08 Hz (Zang et al., 2007). The ReHo value for each voxel was obtained by calculating Kendall’s coefficient of concordance within a cubic cluster size of 27 voxels (Zang et al., 2004). We compared the changes of ALFF and ReHo differences (post-treatment minus pre-treatment) within the group by using paired *t*-test. Results were assumed to be statistically significant at *p* < 0.05 after false discovery rate (FDR) correction within the whole brain. fMRI image processing was carried out using Statistical Parametric Mapping 12 (SPM12^1^) and Data Processing and Analysis for Brain Imaging toolbox version 2.3 (DPABI v. 2.3^2^) software (Yan et al., 2016). The first ten volumes of individual fMRI data were discarded, and slice timing and realignment correction were performed for the remaining images. Any participant with maximum head movement greater than 2.0 mm translation or more than 2.0° rotation was not included. The individual fMRI images were then spatially normalized to the standard template and re-sampled to a 3 mm × 3 mm × 3 mm voxel size. Then, in the regression step, we used multiple regression to model the time-varying BOLD signal in each voxel, including the Friston 24 motion parameters (Friston et al., 1996), cerebrospinal and white matter signals. Afterward, the linear trends were regressed and a band-pass filter was applied at 0.01∼0.08 Hz. Finally, given that resting-state activity is sensitive to minor head movement, we calculated the mean frame-wise displacement (FD) to further determine the comparability of head movement across groups (TA: 0.14 ± 0.06; SA: 0.13 ± 0.04; mean ± SD, *p* = 0.52).

#### Correlation Between Amplitude of Low-Frequency Fluctuations/Regional Homogeneity Values and Clinical Variables

To investigate the correlation between ALFF/ReHo values and clinical variables, Pearson correlation analyses were performed in a voxel-wise manner using DPABI V2.3 software. The correlation analysis was adjusted for the same covariates as those controlled for in the between-group tests. The demographic characteristics of the patients (including age, height, weight) were used as covariates. The statistical threshold was set at *p* < 0.05 (FDR corrected) to explore the most significant correlations among MR voxels.

## Results

### Participants

The demographic characteristics of all subjects are summarized in Table 3. We found no statistical differences between the TA group and the SA group in terms of their age, height, weight, SAS, SDS, frequency of migraine attacks, VAS score, or intensity of the migraine (all *p* > 0.05).

**TABLE 3:** Baseline characteristics of the patients.

Characteristics	TA (*n* = 24)	SA (*n* = 20)	*p*-value
Age (years)	33.04 ± 6.43	35.30 ± 9.43	0.173
Height (cm)	158.68 ± 5.02	159.14 ± 3.99	0.735
Weight (kg)	53.00 ± 6.27	52.90 ± 5.59	0.957
SAS	49.95 ± 10.82	49.96 ± 10.72	0.998
SDS	51.19 ± 12.32	50.36 ± 12.92	0.826
Attack frequency (times)	3.29 ± 1.59	3.76 ± 2.35	0.436
VAS score	6.40 ± 1.55	5.60 ± 1.57	0.095
Intensity of the migraine	2.00 ± 0.54	2.04 ± 0.67	0.829

*Values are expressed as (Mean ± SD).SA, sham acupuncture; SAS, Zung self-rating anxiety scale; SDS, Zung self-rating depression scale; TA, true acupuncture; VAS, visual analog scale.*

*The *p* value was obtained by two-sample *t* test.

### Clinical Outcomes

Comparison within each group, both the TA and SA group showed significant decreases in the SAS, SDS, and VAS scores and intensity of migraine after 12 weeks of treatment (*p* < 0.05). And there was no difference between the two groups in the improvement of clinical symptoms. The frequency of attacks was significantly lower in the TA group than in the SA group (*p* < 0.05). Furthermore, no significant interaction effect was observed between the two groups by analysis of variance for repeated measures in terms of the SAS, SDS, frequency of migraine attack, VAS score every 4 weeks and the intensity of migraines at the end of treatment (all *p* > 0.05) (Table 4).

**TABLE 4:** Clinical outcome measures in each group.

	TA (*n* = 24)	SA (*n* = 20)	*p* ^b^	*p* ^c^
			
Outcome Measure	Mean ± SD	Mean ± SD		
SAS				
Baseline	49.95 ± 10.82	49.96 ± 10.72	0.998	*p*^T^ = 0.000
4 weeks	45.29 ± 7.97	46.68 ± 11.92	0.639	*p*^T*G^ = 0.641
8 weeks	42.52 ± 6.94	45.64 ± 10.19	0.639	*p*^G^ = 0.521
12 weeks	42.81 ± 8.28	45.16 ± 12.28	0.460	–
*p*^a^	<0.001	0.016	–	–
SDS				
Baseline	51.19 ± 12.32	50.36 ± 12.92	0.826	*p*^T^ = 0.000
4 weeks	48.19 ± 11.90	48.24 ± 14.11	0.990	*p*^T*G^ = 0.359
8 weeks	46.09 ± 12.14	47.04 ± 10.45	0.778	*p*^G^ = 0.777
12 weeks	44.29 ± 12.57	46.20 ± 11.41	0.591	–
*p*^a^	0.003	0.005	–	–
Attack frequency (times)				
Baseline	3.76 ± 2.35	3.29 ± 1.59	0.436	*p*^T^ = 0.002
4 weeks	3.44 ± 2.18	4.95 ± 3.58	0.085	*p*^T*G^ = 0.204
8 weeks	3.00 ± 2.04	3.62 ± 3.12	0.424	*p*^G^ = 0.313
12 weeks	2.64 ± 2.89	3.29 ± 1.77	0.378	–
*p*^a^	0.025	0.149	–	–
VAS score				
Baseline	6.40 ± 1.55	5.60 ± 1.57	0.095	*p*^T^ = 0.000
4 weeks	5.44 ± 1.63	4.81 ± 2.16	0.266	*p*^T*G^ = 0.590
8 weeks	4.52 ± 2.38	4.05 ± 2.06	0.480	*p*^G^ = 0.346
12 weeks	3.16 ± 2.24	3.33 ± 1.90^#^	0.782	–
*p*^a^	<0.001	<0.001	–	–
Intensity of the migraine				
Baseline	2.00 ± 0.54	2.04 ± 0.67	0.829	*p*^T^ = 0.000
4 weeks	1.43 ± 0.926	1.96 ± 0.539	0.019	*p*^T*G^ = 0.144
8 weeks	1.38 ± 0.740	1.64 ± 0.995	0.330	*p*^G^ = 0.112
12 weeks	1.38 ± 0.669	1.60 ± 0.913	0.367	–
*p*^a^	0.006	0.019	–	–

*Values are expressed as (Mean ± SD).SA, sham acupuncture; SAS, Zung self-rating anxiety scale; SDS, Zung self-rating depression scale; TA, true acupuncture; VAS, visual analog scale.*

*The *p*^*a*^ based on a paired-*t* test within-group comparisons; The *p*^*b*^ based on *t*-test between the two groups; *p*^*c*^ based on repeated measures; *p*^*T*^, values for comparison between different time points; *p*^T*G^ based on Time*Group interaction; *p*^*G*^ based on comparison between different groups.

### Neuroimaging Results

**FIGURE 1:**
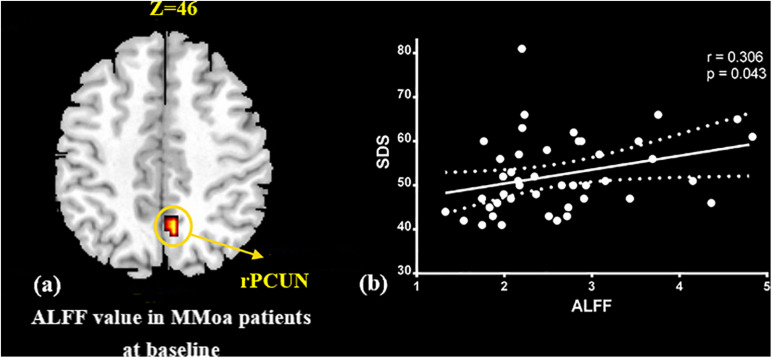
Correlation analysis between ALFF value in precuneus and SDS in the MMoA patients (*p* < 0.05, FDR corrected). **(a)** Brain region used for the correlation analysis shown. **(b)** ALFF in the precuneus was positively correlated with SDS. PCUN, precuneus; R, right; SDS, Zung self-rating depression scale.

**FIGURE 2:**
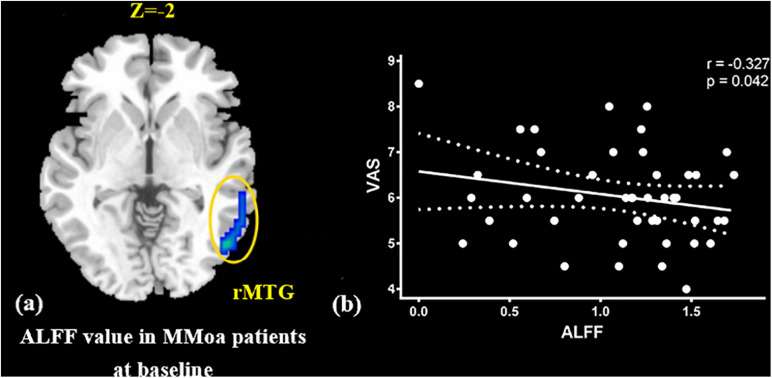
Correlation analysis between ALFF value in MTG and VAS in the MMoA patients (*p* < 0.05, FDR corrected). **(a)** Brain region used for the correlation analysis shown. **(b)** ALFF in the MTG was positively correlated with VAS. MTG, middle temporal gyrus; R, right; VAS, visual analog scale.

**FIGURE 3:**
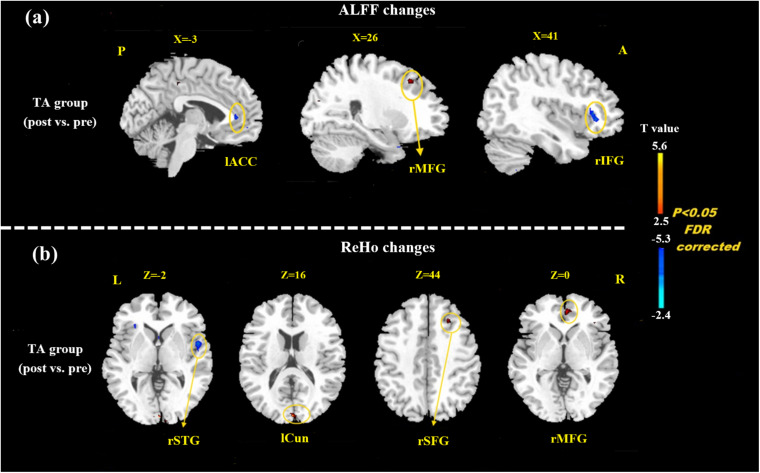
Resting state ALFF and ReHo changes of MMoA patients in true acupuncture group (*p* < 0.05, FDR corrected). **(a)** Brain regions showed altered ALFF value in MMoA patients after true acupuncture treatment. **(b)** Brain regions showed altered ReHo value in MMoA patients after true acupuncture treatment. Warm colors indicate ALFF and ReHo increases after true acupuncture group; cool colors indicate ALFF and ReHo decreases after true acupuncture group. A, anterior; ACC, anterior cingulate; Cun, cuneus; IFG, inferior frontal gyrus; L, left; MFG, middle frontal gyrus; P, posterior; R, right; SFG, superior frontal gyrus; STG, superior temporal gyrus; TA, true acupuncture.

**FIGURE 4:**
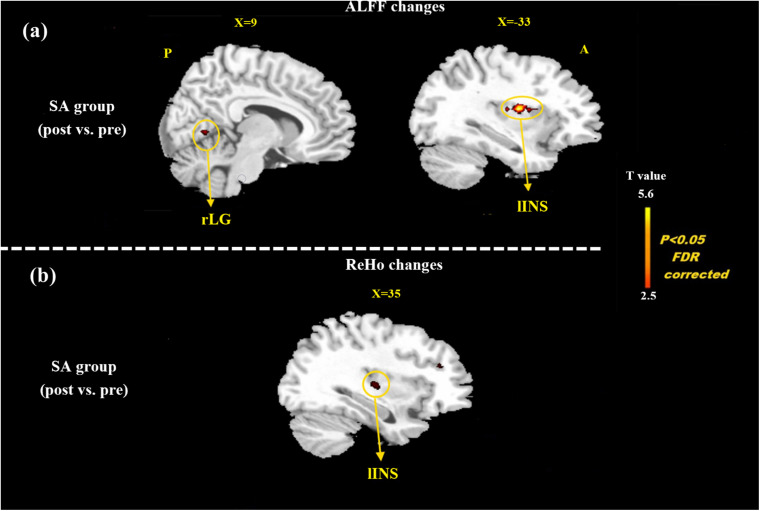
Resting state ALFF and ReHo changes of MMoA patients in sham acupuncture group (*p* < 0.05, FDR corrected). **(a)** Brain regions showed altered ALFF value in MMoA patients after sham acupuncture treatment. **(b)** Brain regions showed altered ReHo value in MMoA patients after sham acupuncture treatment. Warm colors indicate ALFF and ReHo increases after sham acupuncture group; cool colors indicate ALFF and ReHo decreases after sham acupuncture group. A, anterior; INS, insula; LG, lingual gyrus; P, posterior; SA, sham acupuncture.

**FIGURE 5:**
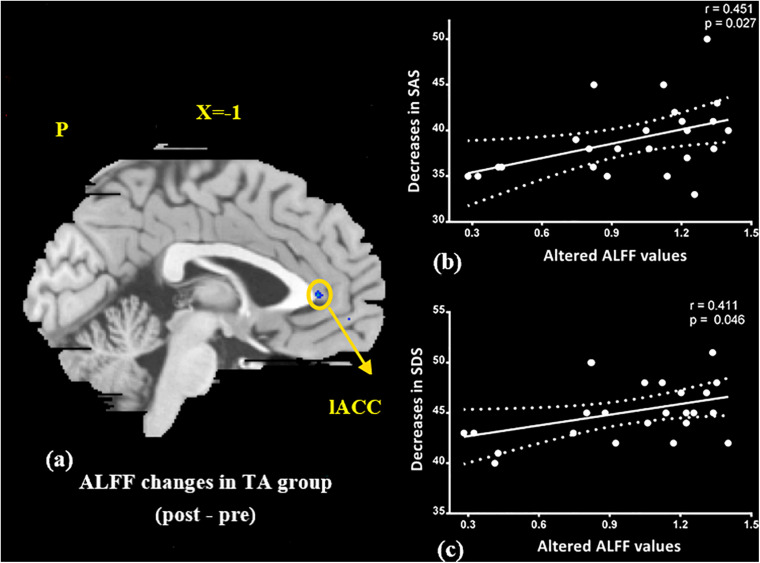
Correlation analysis between altered ALFF values in ACC and improvements in the SAS, SDS in the TA group (*p* < 0.05, FDR corrected). **(a)** Brain region used for the correlation analysis shown. **(b)** ALFF in the ACC was positively correlated with SAS. **(c)** ALFF in the ACC was positively correlated with SDS. ACC, anterior cingulate; L, left; P, posterior; SAS, Zung self-rating anxiety scale; SDS, Zung self-rating depression scale; TA, true acupuncture.

At the baseline, ALFF value in the right precuneus was positively correlated with SDS in the patients with MMoA (*r* = 0.33, *p* < 0.05) (Figure 1 and Table 5), and ALFF value in the right middle temporal gyrus (MTG) was negatively correlated with VAS (*r* = 0.29, *p* < 0.05) (Figure 2 and Table 5). After TA treatment, MMoA patients showed higher ALFF value in the right middle frontal gyrus (MFG); lower ALFF values were observed in the left anterior cingulate (ACC) and right inferior frontal gyrus (Figure 3 and Table 6). Regarding the ReHo values, we found higher ReHo values in the right superior frontal gyrus, left cuneus, and right MFG, while the right superior temporal gyrus showed a lower ReHo value in MMoA patients (Figure 3 and Table 6). After SA treatment, higher ALFF values were observed in the right lingual gyrus and left insula (Figure 4 and Table 7), and left insula showed a higher ReHo value in patients with MMoA (Figure 4 and Table 7). Furthermore, the correlation result showed that the altered ALFF value in the left ACC was positively correlated with the decreases in the SAS scores (*r* = 0.49, *p* < 0.05) and SDS scores in the TA group (*r* = 0.41, *p* < 0.05) (Figure 5). And no significant correlations were observed between brain regions and clinical variables in the SA group.

**TABLE 5:** Baseline ALFF values in patients with MMoA.

Brain region	Hemi	Cluster size	Talairach			Peak *t* value	BA
				
		voxels	x	y	z		
ALFF							
Precuneus	R	48	24	−70	53	7.03	7
Middle temporal gyrus	R	145	59	−58	6	–5.93	21

*ALFF, amplitude of low frequency fluctuations; BA, Brodmann Area; Hemi, Hemisphere; MMoA, menstrual migraine without aura; FDR corrected, *p* < 0.05.*

**TABLE 6:** ALFF and ReHo changes in patients with MMoA after TA.

Brain region	Hemi	Cluster size	Talairach			Peak *t* value	BA
				
		voxels	x	y	z		
ALFF							
Middle frontal gyrus	R	28	33	34	35	3.75	8
Anterior cingulate	L	23	−3	18	18	–4.84	33
Inferior frontal gyrus	R	78	36	21	−1	–3.98	47
ReHo							
Superior frontal gyrus	R	23	15	−11	64	4.67	18
Cuneus	L	21	0	−93	13	6.62	-
Middle frontal gyrus	R	26	21	33	45	3.47	8
Superior temporal gyrus	R	57	48	−25	15	–3.25	41

*ALFF, amplitude of low frequency fluctuations; BA, Brodmann Area; Hemi, Hemisphere; MMoA, menstrual migraine without aura; ReHo, regional homogeneity; TA, true acupuncture; FDR corrected, *p* < 0.05.*

**TABLE 7:** ALFF and ReHo changes in patients with MMoA after SA.

Brain region	Hemi	Cluster size	Talairach			Peak *t* value	BA
				
		voxels	x	y	z		
ALFF							
Lingual gyrus	R	24	9	−79	−4	4.22	18
Insula	L	81	−39	−14	17	3.15	13
ReHo							
Insula	L	29	−29	9	−16	3.70	13

*ALFF, amplitude of low frequency fluctuations; BA, Brodmann Area; Hemi, Hemisphere; MMoA, menstrual migraine without aura; ReHo, regional homogeneity; SA, sham acupuncture; FDR corrected, *p* < 0.05.*

## Discussion

In this study, we focused on the regulatory effects of acupuncture in regulating the emotional disorders in patients with MMoA using neuroimaging. At the baseline, we found the ALFF value in the precuneus was positively correlated with SDS scores in the patients with MMoA (*p* < 0.05). The precuneus is a core region that is responsible for baseline brain activity and participates in functions, such as intrinsic ongoing mental processes and fundamental cognitive social functions (Pereira-Pedro and Bruner, 2016). Furthermore, the precuneus is associated with interoceptive and emotional processing with widely distributed networks sharing connectivity with many brain regions in the frontal, temporal, occipital, and parietal cortices (Kitada et al., 2014). Several neuroimaging studies (Greicius et al., 2007; Sun et al., 2018; Zhu et al., 2018) have reported that depression is associated with abnormalities in precuneus at the resting state. Based on the findings of positive correlation between SDS scores and ALFF in the precuneus, it appears that depressive symptoms may affect the precuneus. The precuneus then enhances its core action on maintaining baseline brain activity, which suggests that the precuneus plays a crucial role in emotional processing of patients with MMoA. The MTG is an associative multisensory area that plays a role in assigning affective tone to short-term memories relating to pain. It also processes visual, olfactory, and auditory sensations (Olson et al., 2007; Asari et al., 2008). These results are in line with those of previous fMRI studies that showed functional alterations in the temporal lobe of patients with migraine (Absinta et al., 2012; Zhao et al., 2013). Our previous fMRI study in female patients with migraine found alterated nodal centrality in the MTG (Liu et al., 2012), and this suggests that long-term and high-frequency headache attacks may lead to pathological cortical network reorganization in female patients with migraine. The correlation analysis results showed that the ALFF values in the MTG were negatively correlated with VAS scores (*p* < 0.05), and this suggest that MTG excitability in form of sensitization during the resting state may contribute to the severity of headache.

Based on the clinical outcomes of this study, both TA and SA helped treat migraine after 12 weeks of therapy (*p* < 0.05). Both treatments remarkably improved the emotional states and alleviated the clinical symptoms of migraine (VAS score and intensity of pain). These results are in line with those of our previous RCT report on migraine (Zhao et al., 2017) and those of previous studies (Smith et al., 2018; Liao et al., 2020). Furthermore, TA was more effective than SA in controlling the frequency of migraine attack, and this has been supported by the findings of several other RCTs (Li et al., 2012; Zhao et al., 2014; Wang et al., 2015).

The clinical effects of SA are similar to those of TA in terms of SAS, SDS, VAS score, and intensity of pain; therefore, we inferred that the clinical effects of SA may partly result from non-specific physiological effect experienced during needling or a placebo effect that originates from frequent patient-acupuncture practitioner interactions (Zhao et al., 2017). Non-specific effects are mostly thought to be due to psychobiological processes triggered by the overall therapeutic context (Finniss et al., 2010). Kong et al. (2006) found that after placebo acupuncture treatment, subjective pain-rating reduction on the placebo-treated side was significantly greater than on the control side, and this demonstrated that the non-specific effect or placebo effect may significantly contribute to the analgesic effect observed during acupuncture (Kong et al., 2013). Additionally, the invasive treatment techniques are almost always perceived by patients as profoundly meaningful, and this could contribute to symptom reduction (Kaptchuk et al., 2006). Taken together, acupuncture is an effective treatment that can improve the emotional state and relieve pain in patients with MMoA, and the current study demonstrates that TA has a relatively better effect in reducing the frequency of migraine attack than SA.

Based on the resting-state fMRI results, regions of the brain that usually respond to TA treatment in terms of both ALFF and ReHo values, were mainly in the frontal-limbic regions, that are associated with regulation of emotions. Previous neuroimaging studies (Fournier et al., 2013; Guo et al., 2015; Li et al., 2020b) have amassed substantial evidence regarding functional abnormalities in fronto-limbic structures. These could be a neurobiological basis for the pathophysiology and maintenance of emotional disorders (Phillips and Kupfer, 2013). Dysfunction in the frontal-limbic regions may reflect impaired high-order frontal regulatory effects on the areas of the limbic system that are related to emotions, and this may lead to mood dysregulation in patients with MMoA. Acupuncture can modulate the activity of cortical and subcortical regions involved in emotional processing (Zhang et al., 2012). The altered ALFF values in the ACC were positively correlated with reductions in the SAS and SDS scores (*p* < 0.05) after TA treatment. ACC is considered to be implicated in both affective and cognitive-attentional dimensions of pain and plays a deterministic role in pain modulation and analgesia (May, 2008).

In our previous neuroimaging studies, we verified that migraineurs showed a significant decrease in ALFF in the ACC (Xue et al., 2013) and showed aberrant functional connectivity involving the ACC (Xue et al., 2012; Yuan et al., 2013). The results of our previous study (Zhao et al., 2014) also suggested that alterations in ReHo values in the ACC might be the mechanism of acupuncture treatment in patients with migraine. In the present study, acupuncture-induced reduction in emotional state was positively associated with decreased average ALFF values in the ACC, which demonstrated that TA treatment could alleviate emotional disorders by modulating emotion-affected dysfunctional regions. Common brain regions that respond to SA treatment both in terms of ALFF and ReHo values are mainly in the insula, and this indicates that SA activates alterations in spontaneous brain activity, resulting in enhanced synchronization. The insula is a major site for processing emotions, and it is also involved in processing sensory discriminative aspects of pain perception (Duerden and Albanese, 2013). Functional imaging experiments have reported that the insular networks were altered in patients with migraine (Maleki et al., 2012), and our team further discovered abnormal patterns of functional connectivity in the insula (Xue et al., 2012; Yuan et al., 2013). However, Wager et al. (2004) found that the placebo effect was achieved through the insula’s regulation of the brain’s sensitivity to pain. Our findings demonstrate that the insula was activated by acupuncture stimulation, and it plays an important role in placebo modulation of pain perception. The insula cortex also plays a reciprocal role in emotions and pain-related emotions. In the current study, the SA group showed significant reductions in the SAS and SDS scores after treatment (*p* < 0.05). We speculated that SA may modulate the perception of pain in patients with MMoA through the insula and indirectly regulate pain-related emotions.

## Conclusion

Our findings reveal that dysfunctions in the precuneus and the frontal-limbic regions may lead to mood dysregulation in patients with MMoA. Furthermore, temporal pole excitability as sensitization may contribute to the severity of headaches. Both TA and SA treatments remarkably improved the emotional states and alleviated the clinical symptoms of migraine. Although there was no difference between the two groups in the improvement of clinical symptoms, but we found that TA had a relatively better effect in controlling the frequency of migraine attacks than SA. Moreover, the effects of the two acupuncture methods on brain activity were significantly different. TA treatment might have the potential effect of alleviating emotional disorders by modulating the frontal-limbic regions, and SA may modulate the perception of pain in patients with MMoA through the placebo effect of the insula and by indirectly regulating emotional disorders.

## Limitation

This study has several limitations. First, because hormonal testing was not carried out, we were unable to compare the hormone levels of patients in the two groups. Considering the important role of hormones in MMoA, hormonal assay should be introduced in future studies to explore the influence of hormones on neuroimaging results. Second, to explore the central mechanisms underlying acupuncture for the treatment of MMoA, we chose not to include a group of healthy subjects as controls at baseline, and this may have prevented us from exploring the pathogenic mechanisms of MMoA in detail. Third, we did not have an index to access and quantify expectations during acupuncture treatment sessions. Further studies need to quantify patients’ expectations and explore the effect of acupuncture on the clinical efficacy and physiological mechanism of some non-specific factors during long-term acupuncture treatment.

## Data Availability Statement

The raw data supporting the conclusions of this article can be obtained on reasonable request from the corresponding author. Requests to access the datasets should be directed to LZ, zhaoling@cdutcm.edu.cn.

## Ethics Statement

The studies involving human participants were reviewed and approved by Sichuan Traditional Chinese Medicine Regional Ethics Review Committee (approval number: 2015KL-004). The patients/participants provided their written informed consent to participate in this study.

## Author Contributions

YTZ and LZ: conception and study design. ZWW, JRD, WY, YZ, HQL, and DHL: data collection or acquisition. YTZ, TX, and XW: statistical analysis. YTZ and ZWW: drafting the manuscript work. LZ: revising it critically for important intellectual content. All authors contributed to the article and approved the submitted version.

## Conflict of Interest

The authors declare that the research was conducted in the absence of any commercial or financial relationships that could be construed as a potential conflict of interest.

## Publisher’s Note

All claims expressed in this article are solely those of the authors and do not necessarily represent those of their affiliated organizations, or those of the publisher, the editors and the reviewers. Any product that may be evaluated in this article, or claim that may be made by its manufacturer, is not guaranteed or endorsed by the publisher.

## Footnotes

1
https://www.fil.ion.ucl.ac.uk/spm/

2
http://rfmri.org/dpabi

## Abbreviations

ACC: anterior cingulate
ALFF: amplitude of low-frequency fluctuation
BOLD: blood oxygen level-dependent
CI: confidence interval
fMRI: functional magnetic resonance imaging
ICHD-3: third edition of the International Classification of Headache Disorders
MFG: middle frontal gyrus
MMoA: menstrual migraine without aura
MTG: middle temporal gyrus
RCT: randomized clinical trial
ReHo: regional homogeneity
SA: sham acupuncture
SAS: Zung self-rating anxiety scale
SDS: Zung self-rating depression scale
TA: true acupuncture
TCM: traditional Chinese medicine
VAS: visual analog scale.
